# Electrochemically decoupled reduction of CO_2_ to formate over a dispersed heterogeneous bismuth catalyst enabled *via* redox mediators†

**DOI:** 10.1039/d3ey00271c

**Published:** 2023-11-28

**Authors:** Mark Potter, Daniel E. Smith, Craig G. Armstrong, Kathryn E. Toghill

**Affiliations:** a Department of Chemistry, Lancaster University Lancaster LA1 4YB UK k.toghill@lancaster.ac.uk

## Abstract

Electrochemical CO_2_ reduction is a topic of major interest in contemporary research as an approach to use renewably-derived electricity to synthesise useful hydrocarbons from waste CO_2_. Various strategies have been developed to optimise this challenging reaction at electrode interfaces, but to-date, decoupled electrolysis has not been demonstrated for the reduction of CO_2_. Decoupled electrolysis aims to use electrochemically-derived charged redox mediators – electrical charge and potential vectors – to separate catalytic product formation from the electrode surface. Utilising an electrochemically generated highly reducing redox mediator; chromium propanediamine tetraacetate, we report the first successful application of decoupled electrolysis to electrochemical CO_2_ reduction. A study of metals and metal composites found formate to be the most accessible product, with bismuth metal giving the highest selectivity. Copper, tin, gold, nickel and molybdenum carbide heterogeneous catalysts were also investigated, in which cases H_2_ was found to be the major product, with minor yields of two-electron CO_2_ reduction products. Subsequent optimisation of the bismuth catalyst achieved a high formate selectivity of 85%. This method represents a radical new approach to CO_2_ electrolysis, which may be coupled directly with renewable energy storage technology and green electricity.

## Abstract 2

Electrochemical CO_2_ reduction is a topic of major interest in contemporary research as an approach to use renewably-derived electricity to synthesise useful hydrocarbons from waste CO_2_.

## Graphical abstract

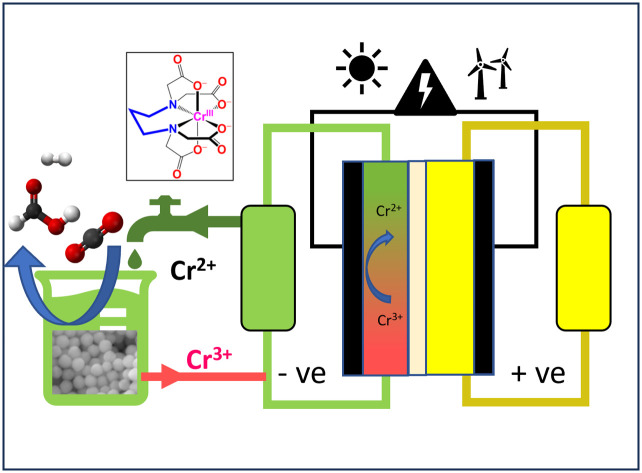

**Broader context**

Efficient and widespread utilisation of renewable energy remains a crucial component of decarbonisation, to which energy storage technology will play an increasingly important role as fossil fuels are phased out of existing systems. Furthermore, electrochemical synthesis of commodity chemicals offers a promising alternative to those derived from the petrochemical industry. As a consequence of their intermittent nature, wind and solar power require coupling with energy storage so as not to introduce instability within the existing power network. Amongst the existing technologies, batteries offer the best solution to the enormous scale that is required. Nevertheless, even the most well-designed system will have a finite capacity, after which surplus energy produced during peaks times will be wasted. Developing hybrid energy storage technologies, such as by combing electrical energy storage with chemical energy storage through electrochemical reactions, will allow for utilisation of this otherwise wasted energy.

## Introduction

### Background

Fossil fuels, as a source of energy and chemical feedstocks, have been and remain critical to the success and prosperity of humanity, but somewhat ironically their perpetual consumption has given rise to considerable pollution of which most concerning is the vast quantity of the greenhouse gas, carbon dioxide. CO_2_ continues to have a encapsulating radiative effect on the Earth's climate, leading to a steady increase in average global temperature and ocean acidification.^1^ Unfortunately, the intermittent, weather-dependent nature of the most accessible renewable energy sources introduces supply concerns when relied upon to contribute a large portion of the electrical grid, necessitating both short- and long-term energy storage solutions. A wide portfolio of energy storage technologies is being developed, including high-power supercapacitors, high-capacity batteries and long-term chemical storage.

The leading battery technologies, lithium-ion and sodium–sulphur, provide large-scale grid storage with high energy density, although their power and capacity are intrinsically linked and they require strict thermal management.^2–5^ In contrast, redox flow batteries (RFBs) present an alternate technology uniquely suited to large-scale stationary storage. While their energy density is much lower than that of other technologies, their power and capacity can be scaled independently and tailored to the requirements of the application, by increasing electrode area or electrolyte volume respectively.

Energy can also be stored chemically by using electrical energy to drive otherwise unfavourable reactions *via* an externally applied potential. The simplest of these reactions is the electrolysis of water to hydrogen and oxygen, which is already commercialised through an array of technologies such as proton exchange membrane (PEM) and alkaline water (AWE) electrolysers,^6^ although large-scale adoption is hindered by high cost.^7^

### Redox mediators and decoupled electrolysis

An emerging technique in electrochemistry is the use of reversible redox species as charge transfer mediators to boost the performance of existing systems. This includes the incorporation of redox mediators in solid-state battery technologies as secondary charge carriers to improve electron transfer efficiency and increase energy density,^8,9^ and their use as co-factor analogues to boost the performance of molecular catalysts.^10–12^

In 2013, a new approach to water splitting emerged in which the redox mediator plays the pivotal role, time decoupled electrolysis, where an electrochemically-generated redox mediator is used to store charge and energy. The concept was introduced by Symes and Cronin, where they used the polyoxometalate phosphomolybdic acid as an ‘electron-coupled proton buffer’ to store protons and electrons released during water oxidation and release them during water reduction.^13^ Decoupling the reaction in this way lowers the energy requirement at any given time by disconnecting the half reactions for H_2_ and O_2_ evolution and linking them to the intermediate potential of the redox mediator such that the each of the two individual reactions are less energy demanding than direct water splitting. This has the added benefit of removing the intrinsic rate-limiting link between the facile proton reduction reaction and the much slower water oxidation reaction, such that the two processes are not forced to proceed at the same rate and are instead linked to the rate of electron transfer to/from the mediator. In principle, any electrolytic reaction can be decoupled in this way.

The concept of the ‘dual circuit flow battery’ was simultaneously developed by the Girault group, where a vanadium–cerium redox flow battery was adapted to produced hydrogen and oxygen *via* water splitting in a spatially decoupled manner by chemically discharging the electrolytes over catalytic beds.^14^ The system effectively conveyed the proof of concept, however the Ce(iii)/Ce(iv) redox coupled limited the energy efficiency and required a highly corrosive electrolyte. The system was then further developed by swapping the Ce couple with Mn(ii)/Mn(iii), resulting in a more stable system capable of releasing stored energy both electrically and chemically, with enhanced product purity over conventional electrolysers owing to the spatial separation of product formation.^15^ Decoupling the reaction in this way requires matching of the mediator's redox potential with the onset of the electrochemical reaction.

**Fig. 1:**
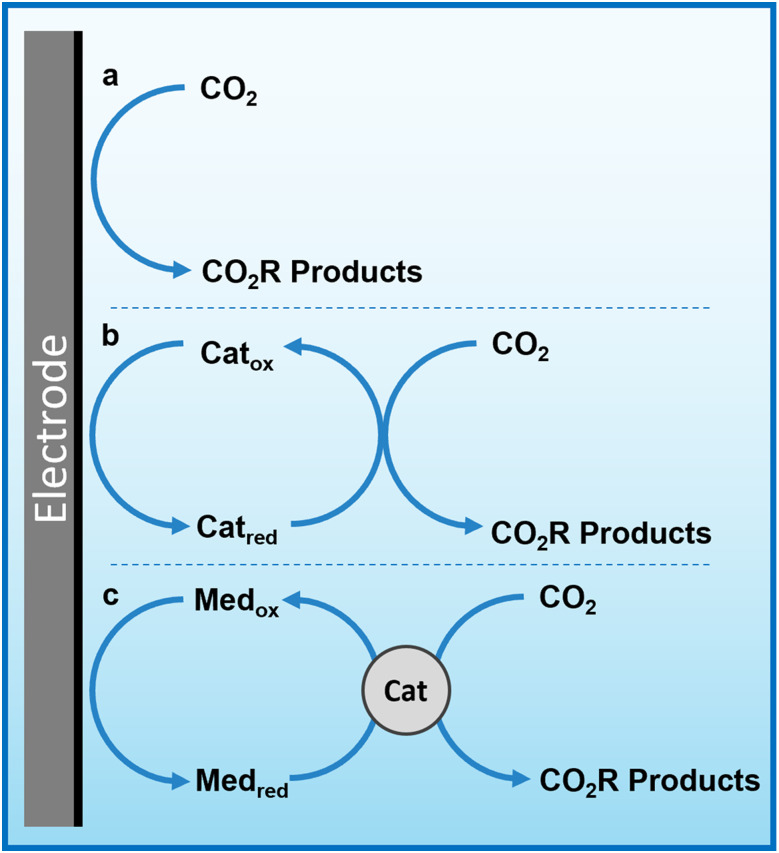
Simplified mechanism of heterogeneous, homogeneous and redox mediated heterogeneous electrochemical CO_2_ reduction. (a) Conventional heterogenous electrocatalysis, in which electrons are transferred from the electrode to CO_2_ molecules on/near the electrode surface. (b) Homogeneous (or molecular) electrocatalysis, in which redox-active catalyst molecules are reduced at the electrode surface and subsequently react with CO_2_ in solution. (c) Redox mediated electrocatalysis, in which a redox-active species is reduced at the electrode surface and subsequently transfers this charge and potential energy to a catalyst that then electrochemically reduces CO_2_.

Herein, we propose that another important and widely-studied reaction, electrochemical CO_2_ reduction (ECO_2_R), can be decoupled in a similar manner. In this instance, decoupled refers to the spatial decoupling of the electrocatalytic reaction from the electrode surface, as show in Fig. 1c. By applying a decoupled approach, it is hoped that the limitations of a conventional heterogeneous electrode-catalyst – limited surface area, low concentration of dissolved CO_2_ at the electrode, and the need for high electrical conductivity – can be overcome. Study and enhancement of the ECO_2_R remains a fundamentally challenging endeavour, still plagued by selectivity, activity and efficiency limitations long after its conceptualisation. From the initial work by Hori with simple monometallic electrodes in the 1980s,^16^ to the latest nanostructured, highly-tailored catalysts designed with the aid of computational methods, no catalyst has managed to achieve economic viability.^17^ Copper remains the front-runner, capable of producing a range of low-carbon species, most notably methane and ethylene, albeit with poor selectivity and stability.^18–20^ Most other catalysts are limited to the two-electron products of formic acid or carbon monoxide.^21^ It is envisioned that decoupling catalysis from the electrode surface will widen the scope of viable catalyst materials, possibly with improved performance. Herein we report the first demonstration of a decoupled electrochemical CO_2_ reduction (DECO_2_R) reaction, in which the HER is suppressed to increase selectivity towards the production of formate.

## Experimental

### Chemicals and reagents

All chemicals were used as purchased without further purification. Chromium potassium sulfate dodecahydrate, sodium chloride (99%), potassium hydrogen carbonate (99.7%), potassium ferrocyanide (99%), bismuth powder (99.5%, 100 mesh), copper powder (99.5%, 100 mesh), tin powder (99.5%, 100 mesh), acetone (99%), and 1-octadecene (90% technical grade) were purchased from Thermo Fisher Scientific (Alfa Aesar, Acros Organics). 1,3-Propanediamine-*N*,*N*,*N*′,*N*′-tetraacetic acid (99%), 1-octanethiol (98.5%), oleylamine (70% technical grade), bismuth neodecanoate, bismuth nitrate pentahydrate (99.99%), ethylene glycol (99%), and molybdenum carbide were purchased from Merck (Sigma Aldrich). Isopropanol (99.5%) was purchased from Honeywell. Potassium hydroxide (reagent grade) was purchased from VWR. Gold mesh (99.9%, 0.06 mm diameter, 20 × 20 mm area, 82 wires per inch) and bismuth foil (99.999%, 0.25 × 10 × 10 mm) were purchased from Goodfellow Cambridge Ltd.

### Electrochemical methods

Electrochemical experiments were performed using a Biologic SP300 potentiostat. Voltammetric characterisation was performed in a standard three-electrode cell using a 3 mm diameter glassy carbon disk working electrode, Pt coil counter electrode and an Ag/AgCl reference electrode. Cyclic voltammetry was performed at a scan rate of 100 mV s^−1^ unless otherwise stated. Electrolyte charging was conducted in a custom flow cell (Fig. S20, ESI†) with an electrode surface area of 16 cm^2^, utilising GFD 4.6 SIGRACELL graphite felt electrodes heat-treated for 4 hours at 500 °C under air to improve hydrophilicity, and a fumapem® F-930 cation exchange membrane.

### Synthesis

Potassium chromium 1,3-propanediamine-*N*,*N*,*N*′,*N*′-tetraacetate was synthesised in a single step reaction from readily available precursors. 1,3-Propanediamine-*N*,*N*,*N*′,*N*′-tetraacetic acid (9.18 g) and potassium chromium sulfate dodecahydrate (14.48 g) were added to a round bottom flask (RBF) along with water (60 mL), and the mixture was refluxed for 2 hours. Potassium hydroxide (6 g) was added and the mixture was stirred for a further hour, followed by dropwise addition of potassium hydroxide solution (5 M) until the mixture was fully neutralised. An equal volume of acetone (approx. 100 mL) was then added, causing a pale purple precipitate to form, which was removed by vacuum filtration. The filtrate was reduced under vacuum to approx. 60 mL and then added dropwise by pipette to ice cold isopropanol (300 mL), resulting in a pink precipitate that was collected by vacuum filtration and washed with isopropanol. CHNS elemental analysis indicated the presence of a small sulfate impurity (2–3%), along with 1–2 waters of crystallisation. Measured: (6.77% N, 31.02% C, 4.12% H, 0.22% S), expected: (7.12% N, 33.60% C, 3.59% H, 0.00% S).

Bismuth nanospheres (hereafter referred to as Bi NS) and bismuth rhombic dodecahedron nanoparticles (hereafter referred to as Bi RD) were synthesised by methods adapted from existing literature.^22^ Bi NS: bismuth nitrate pentahydrate (4.55 g) was added to a 125 mL PTFE lined autoclave (Parr model 4748) along with ethylene glycol (37.5 mL), which was sealed and heated to 180 °C for 24 hours. The resulting nanoparticles were washed by centrifugation and redispersion in ethanol, then water three times each; and were dispersed in water (80 mL) and stored as an ink with an approximate loading of 19 g L^−1^. Bi RDNP: bismuth neodecanoate (720 mg) and 1-octadecene (5 mL) were added to an argon-flushed RBF and heated at 85 °C for 30 min, after which 1-octanethiol (10 mL) was injected, causing the mixture to turn yellow/brown. This was heated for a further 30 min, followed by the addition of olyelamine (10 mL) and another 30 min of heating, turning the mixture black and opaque. Once cooled, the resulting nanoparticles were washed by centrifugation and redispersion in ethanol three times. The morphology of the nanoparticles was subsequently confirmed by scanning electron microscopy (Fig. S9, ESI†).

### Catalyst screening

Batch decoupled reduction reactions were performed in a sealed Schlenk tube, evacuated and replenished with CO_2_ five times to ensure a controlled atmosphere. The redox mediator solution was charged in the custom flow cell against an excess of K_4_[Fe(CN)_6_] under an inert N_2_ atmosphere. Once charged, the solution was saturated with CO_2_ and a 20 mL aliquot was injected into the reaction vessel containing the catalyst material and was left to react with the aid of magnetic stirring. The reaction was considered complete when the colour of the mediator solution returned to its ground state and ceased effervescing. The catalyst loading was approximately 25 mg for Bi, Cu, Sn, Mo_2_C and Ni powders, 142 mg for the Au mesh, 30 mg for Bi foil, 19 mg for Bi NS, and 10–15 mg for BI RD. Between experiments, the reactor was cleaned mechanically and with concentrated acid to remove residual catalyst.

### Product analysis

Reduction products were analysed by a combination of gas chromatography (GC) for the gas phase and ion chromatography (IC) for the liquid phase. GC was performed utilising a Shimadzu 2030 equipped with a dielectric-barrier ionisation detector (BID) and ResTek Shin Carbon ST 80/100 column. The instrument was calibrated in the 0–1000 ppm range for the gaseous products H_2_, CO, CH_4_ and C_2_H_4_ by use of calibration gas supplied by BOC Ltd. Samples were extracted from the vessel and introduced *via* a gas tight syringe, typically after a 5× dilution to bring the concentrations into the calibration range. IC was performed using a Metrohm Eco IC equipped with a Metrosep organic acids column (250/7.8) and a conductivity detector. Formate concentration was determined from a calibration curve in the 0–100 ppm range by use of a 1000 ppm calibration standard purchased from Sigma Aldrich.

## Results and discussion

### Redox mediator characterisation

**Fig. 2:**
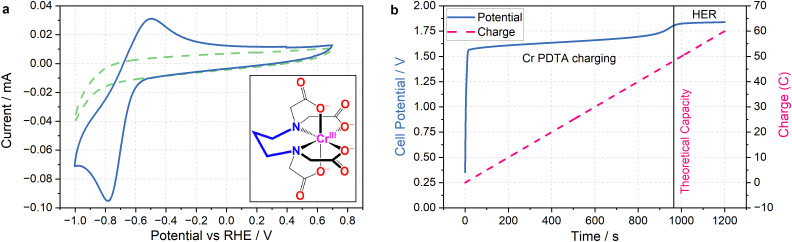
Cyclic voltammetry and charging profile of Cr PDTA. (a) (main) Cyclic voltammograms recorded in 1 M KHCO_3_ supporting electrolyte in water with (solid blue) and without (dashed green) 10 mM Cr PDTA using a glassy-carbon electrode (scan rate of 100 mV s^−1^). Peak separation of 285 mV with broad peaks indicates a quasi-reversible redox process with slow kinetics. (inset) Chemical structure of Cr(iii) PDTA. (b) Charging profile of 10 mM Cr PDTA with 1 M KHCO_3_ supporting electrolyte against excess K_4_Fe(CN)_6_ at 50 mA (electrode surface area 16 cm^2^, Sigracell GFD 4.6). The potential (solid blue) and charge passed (dashed pink) are plotted with respect to time, with the line at *x* = 965 s indicating the theoretical capacity (48.25 C) of the 50 mL of 10 mM Cr PDTA charged during the experiment.

The primary challenge in achieving DECO_2_R is identifying a mediator able to provide sufficient driving force for the less-than-facile reaction. When targeting hydrogen evolution, a mediator simply needs a redox potential below 0 V *vs.* the reversible hydrogen electrode (RHE) to provide a thermodynamic driving force, whereas most CO_2_ reduction catalysts require operating potentials at least as negative as −0.7 V *vs.* RHE to activate the reaction. Even on relatively inert carbon cloth electrodes, this is already pushing the limits of the water's electrochemical stability, which makes finding aqueous species with sufficiently negative redox processes challenging, and introduces the risk of competition from hydrogen evolution. Fortunately, the wellspring of existing literature detailing RFB electrolytes offers an excellent foundation from which to draw inspiration when selecting a redox mediator. The fully chelated chromium complex of 1,3 propanediamine-*N*,*N*,*N*′,*N*′-tetraacetic acid has recently been highlighted as an aqueous RFB anolyte with an unusually negative single electron reduction at *E*^0^ = −1.1 V *vs.* the standard hydrogen electrode (SHE) (−0.6 V *vs.* RHE in 1 M KHCO_3_ electrolyte), along with high solubility and long-term stability.^23^ As seen in Fig. 2a, the complex operates at the edge of the potential window for aqueous electrochemistry at this pH (8.5), allowing for the construction of aqueous RFBs with large cell potentials, and providing a large driving force for aqueous reduction reactions when used as a redox mediator. The fully chelated nature of the complex should inhibit it from acting as a molecular catalyst, allowing it to effectively transfer charge and energy without undergoing any self-discharge reactions. Indeed, the mediator does not spontaneously discharge while saturated with CO_2_, and an oxidative back-peak is visible in cyclic voltammograms of Cr PDTA saturated with CO_2_ (Fig. S3 and S4, ESI†).

1 Cr(iii) PDTA + e^−^ → Cr(ii) PDTA *E*^0^ = −1.1 V

2 CO_2_ + e^−^ → CO_2_˙^−^ *E*^0^ = −1.9 V

3 CO_2_ + 2H^+^ + 2e^−^ → HCOOH *E*^0^ = −0.61 V

4 CO_2_ + H_2_O + 2e^−^ → HCOO^−^ + OH^−^ *E*^0^ = −0.43 V

5 CO_2_ + 2H^+^ + 2e^−^ → CO + H_2_O *E*^0^ = −0.53 V

6 CO_2_ + H_2_O + 2e^−^ → CO + 2OH^−^ *E*^0^ = −0.52 V

7 2H^+^ + 2e^−^ → H_2_ *E*^0^ = −0.41 V

All *vs.* SHE at pH 7.

The combination of a large driving force and a near neutral pH gives Cr PDTA a large potential over the thermodynamic requirement (overpotential) to act as a redox mediator for ECO_2_R (eqn (2)–(6)).^24^ This driving force also easily enables hydrogen evolution (eqn (7)), which acts as a parasitic competing reaction during charging, even on largely inert carbon felt electrodes and glassy carbon surfaces, and especially if trace metal impurities are present in the electrolyte. This is highlighted by the two distinct regions in Fig. 2b. The first linear potential region between 20 and 900 seconds follows a shallow gradient as controlled by the ratio of Cr(ii)/Cr(iii) according to the Nernst equation (as the K_4_Fe(CN)_6_ is in excess it has little influence over the cell potential). The second linear region between 1000–1200 has minimal gradient as hydrogen evolution has become the dominant electrochemical process and occurs under a steady state. The small potential step between these two processes indicates how close to the edge of the solvent window the mediator operates. It was also noted by Robb *et al.* when reported as a flow battery negolyte that hydrogen evolution was observed on stainless steel fittings and from trace metal impurities in the electrolyte.^23^

### Aqueous decoupled CO_2_ reduction on bulk metal catalysts

To determine the effectiveness of Cr PDTA as a redox mediator for DECO_2_R, a range of simple catalysts were tested to target all possible products. As an initial proof-of-concept, inexpensive bulk metal powders were screened as catalysts using a Schlenk tube as rudimentary batch reactor to establish whether any CO_2_ reduction products could be detected. Metal selection was based on the seminal systematic exploration reported by Hori.^25^ Bismuth and tin powders were used to target formate production, while a reusable gold mesh was used to target CO formation. Copper powder was screened in the hope of observing further reduced products such as methane and ethylene. For comparison, molybdenum carbide and nickel powders were used as expected hydrogen evolution catalysts.

**Fig. 3:**
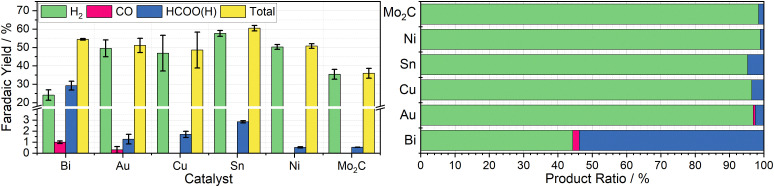
Left: Column chart indicating the average faradaic yields of H_2_ (green), CO (pink), HCOO(H) (blue), and in total (yellow) measured when 20 mL aliquots of 10 mM Cr PDTA were discharged over the heterogenous catalysts Bi, Au, Cu, Sn, Ni, and Mo_2_C inside a sealed CO_2_ atmosphere, with error bars indicating the standard deviation within each triplicate set. Right: Row chart indicating relative product ratios for Cr PDTA mediated CO_2_ reduction over simple heterogeneous catalysts.

Using a fixed volume of charged mediator solution at a known concentration, the total charge passed during the CO_2_ conversion can be estimated, allowing for a determination of the faradaic efficiency of each substituent product by gas and ion chromatography quantification. These results are summarised in Fig. 3. Complete charging is signified by a colour change from the red ground state to the pale green reduced state, and when the charging curve deviates from the plateau region (Fig. 2b).

Bismuth was chosen due to its large overpotential requirement with respect to hydrogen evolution and having been previously observed to reduce CO_2_ almost exclusively to formate.^26^ When used as the catalyst for DECO_2_R mediated by Cr PDTA, bismuth gave the highest yield of CO_2_R products, with formate making up 54 ± 4.7% of the observed products. The remaining 46 ± 4.7% was predominantly H_2_, though trace CO was also detected. The overall faradaic yield when using bismuth was 54 ± 0.4%, with the reaction taking about 30 minutes. While Au is typically considered a good catalyst for CO production,^27^ when used for DECO_2_R mediated by Cr PDTA, only a small amount was observed, with a faradaic yield of just 0.6 ± 0.3%, making up about 1 ± 0.5% of the measured products. Formate accounted for another 2 ± 1% of the products and H_2_ made up the remaining 97 ± 1.6%, with an overall faradaic yield of 50 ± 4.6%. The reaction took around one day to complete. While the bismuth was, along with all the other simple catalysts, a powder consisting of 150 micron or smaller particles, it is important to note that the gold mesh was by comparison macroscopic, and as such had a much lower surface area despite the larger mass deployed (142 mg *vs.* 25 mg).

No further reduction products were observed when using Cu powder as the catalyst, with H_2_ again making up 96 ± 0.8% of the observed products. Formate constituted the majority of the remaining 4 ± 0.8%, with a trace of CO also detected, with a total faradaic yield of 49 ± 9.8%. The reaction appeared complete after 10 minutes. Furthermore, when left in contact for prolonged periods, copper metal displaces the Cr(iii) redox mediator from its chelating ligand, ultimately resulting in a deep blue solution of Cu(ii) (Fig. S18 and S19, ESI†). Sn powder, which was expected to produce formate, also generated mostly hydrogen at 95 ± 0.3% selectivity along with 5 ± 0.3% formate and a trace of CO, with a faradaic yield of 61 ± 1.5%. Again, the reaction took about 10 minutes. As expected Mo_2_C and Ni produced mainly H_2_, at 98.5 ± 0.09% and 98.9 ± 0.08% of the observed products respectively, with overall faradaic yields of 36 ± 2.7% and 51 ± 1.2%. Molybdenum carbide was reasonably active, with discharge taking about 15 minutes, while the nickel displayed poor activity, with discharge taking approximately one day. Recently, molybdenum carbides have been explored as potential catalysts for CO_2_R, both electrochemically and through hydrogenation, however minimal CO_2_R products were observed when used in the Cr PDTA mediated system.^28,29^

Catalyst free control reactions indicated the presence of contamination within the reaction vessel, despite cleaning with aqua regia and ultrapure water between experiments. The charged mediator should be stable under inert conditions, however it appeared to discharge very slowly in the absence of catalyst. The mediator is expected to spontaneously reduce oxygen on contact with air, however product analysis also indicated the formation of a small amount of both H_2_ (13.9 ± 2.2%) and HCOO^−^ (3.9 ± 0.7%) as the mediator discharged passively. Compared to active catalysis, this discharge is very slow and occurred over many days, however it was envisioned that the charged mediator would be stable for long term storage as a battery negolyte. It is suspected that the discharge is caused by metal ion contamination, either from trace impurities within the electrolyte, as has been previously reported, or residual catalyst contamination within the reactor.^20,30^ Under the reducing conditions, these metal ions can be deposited, creating catalytic sites to further discharge the mediator. Equally, it may not be impossible for the mediator to act as a molecular catalyst, albeit very poorly, as the chelating ligand would need to relinquish one or more coordination sites to allow coordination of the reaction intermediates. This introduces concerns when considering the possible effects on long term catalyst stability and selectivity, and raises questions about what portion of the products are truly formed on the intended catalyst compared to the contamination, especially on the less active catalysts. It is likely this contamination contributed towards the large standard deviation seen in the product selectivity of some of the catalysts tested.

### Faradaic losses

The faradaic yields observed were typically in the 50–60% range, indicating some inefficiency in the batch methodology whereby charge is lost to a number of unobserved processes. The first is the reduction of O_2_, for which the charged mediator has considerable driving force. While care was taken to ensure the mediator remained under N_2_ or CO_2_ atmospheres during charging and transfer, it is unavoidable that some charge will be lost due contact with O_2_ from residual air, which was always observed in the GC analysis. It also assumed that the mediator is at a 100% state of charge from the colour change and charging profile, however this is not quantitative. Furthermore, it is possible that gaseous products are lost during transportation and dilution of the samples prior to GC analysis.

A further loss of charge occurs due to a ‘tax’ of electrons that are required to bring the Fermi level of the catalyst in line with the electrochemical potential of the mediator solution when they are introduced, where the particles effectively behave as a series of redox couples. This has been described in detail for small nanoparticles, where the effects of quantisation result in discrete energy levels.^31^ Ultimately, the number of electrons needed to shift the Fermi level will be dependent on a range of factors including particle surface area, number of atoms, and surface species including capping agents and counter ions, as well as the initial Fermi level of the particle, all of which will be unique to each material. For larger particles where the density of energy levels is more akin to bulk metal, a large but finite number of electrons will be transferred in order to bring the system into equilibrium; this assumes the particles are inert, however, as they are acting as electrocatalysts, this is not the case. Instead, a steady state will be established where electrons are transferred onto the particles at the same rate that they are used in reduction reactions. As the reaction proceeds, the Fermi level of the system will shift until there is no longer a driving force and equilibrium is reached. Exploring these effects is far beyond the scope of this initial proof of concept, and as such they are simply taken as a limitation of the batch technique used to screen the catalysts.

### The effect of buffers and pH changes

Interestingly, when the mediator solution and reaction vessel were saturated with N_2_ instead of CO_2_, for the purpose of control experiments, formate was still observed as a product for many of the catalysts, with bismuth powder yielding H_2_ and HCOO^−^ at faradaic efficiencies of 27% and 7.5% respectively; this is coupled with a large peak in the GC trace attributed to CO_2_. The source of this CO_2_ is the KHCO_3_ electrolyte, which is decomposing to fulfil the equilibrium between aqueous carbonate species, releasing CO_2_ which is then accessible for reduction.^25^ It is this fast exchange which makes carbonate electrolytes the favoured choice for ECO_2_R, as it effectively increases the activity of aqueous CO_2_ far beyond the saturation point of dissolved gas, aiding in mass transport to the catalyst surface and providing a buffering effect against localised pH change during electrolysis. Furthermore, there is growing evidence that various dissolved carbonate species are reduced at the electrode surface and contribute to the overall ECO_2_R reaction.^32^

In an effort to fully eliminate CO_2_ from the control experiments, the supporting electrolyte was switched to KCl. This resulted in hydrogen as then only detected product on the bismuth powder catalyst, at a faradaic efficiency of 12%. This change in electrolyte composition had no visible effect on the complex in its ground state, however once charged the mediator solution exhibited a pale blue colouration (Fig. S17b, ESI†). When subsequently discharged, either by a catalyst or by air, the mediator solution no longer returned to its red colour, instead taking on a deep purple hue (Fig. S17c, ESI†). To rationalise the changes in colour, UV/Vis spectra of the mediator were taken for the range of observed states (Fig. S5–S7, ESI†). In its fresh red state, the mediator solution exhibits two strong absorption peaks centred around 510 and 386 nm, with molar absorption coefficients of 89.2 M^−1^ cm^−1^ and 66.6 M^−1^ cm^−1^ respectively. Once charged, neither the green nor blue states display absorbance peaks, but rather have broadly low absorbance above 400 nm and high absorbance below 400 nm. In its spent (purple) state, the mediator again displays two absorbance peaks, slightly redshifted as compared to the fresh solution with the longer wavelength peak much broader and lower in absorbance intensity.

As hydroxide ions are an expected product of all reduction half reactions at a neutral pH (see eqn (4) and (6)), and without any balancing oxidation, the pH of the mediator solution will increase when used in a batch reaction. In its fresh state, with 1 M KHCO_3_ as the supporting electrolyte, the pH was around 8.5, and once charged and spent the pH increased to around 9.2, suggesting the carbonate equilibrium is somewhat able to buffer against pH change. When 1 M KCl was instead used as the supporting electrolyte, the pH of fresh solution was 7.8, and once spent this increased to around 11. A small amount of 1 M HCl was added to return the pH to that of the fresh solution, which restored the red colouration, and the position and ratio of the peaks in the UV/Vis spectrum to that of the fresh solution (Fig. S7, ESI†).

### Optimised bismuth materials as potential catalysts

Having observed bismuth 100-mesh powder as giving the best yields of a CO_2_R product, it was chosen as the focus in preparation of tailored decoupled CO_2_R catalysts. A particularly promising material was reported by Xie *et al.*, where they developed facet-controlled bismuth rhombic dodecahedra with a much less negative onset potential for ECO_2_R compared to bulk metal, with selectivity starting at 85% and peaking at 95% across the −0.6 to −1.2 V *vs.* RHE potential range.^22^ This was compared to both Bi foil and Bi nanospheres, which they found to have selectivity starting around 60% at −0.6 V *vs.* RHE, only increasing to 85% at −0.8 V *vs.* RHE for the nanospheres and −0.9 V *vs.* RHE for the bulk foil, with both achieving a peak selectivity of around 90%. The Cr PDTA mediator operates at the lower end of this scale, where the bulk foil material only achieved a selectivity of around 60% in Xie's work, which compares well with the 55% selectivity we observed in the decoupled system when dispersed bismuth powder was deployed as the catalyst.

**Fig. 4:**
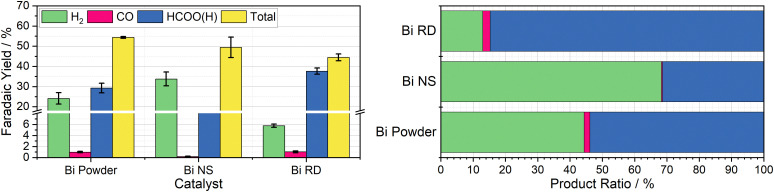
Left: Column chart indicating the average faradaic yields of H_2_ (green), CO (pink), HCOO(H) (blue), and in total (yellow) measured when 20 mL aliquots of 10 mM Cr PDTA were discharged over commercial bismuth powder (50 μm), bismuth nanospheres (50–500 nm), and bismuth rhombic dodecahedra (80 nm) inside a sealed CO_2_ atmosphere, with error bars indicating the standard deviation within each triplicate set. Right: Row chart indicating relative product ratios for Cr PDTA mediated CO_2_ reduction over bismuth catalysts.

Both the rhombic dodecahedra and the nanospheres were synthesised using the procedures described by Xie *et al.* for performance comparison, with the results summarised in Fig. 4. The Bi nanospheres (Bi NS) were synthesised by solvothermal reduction of bismuth nitrate in ethylene glycol. Surface morphology was confirmed by SEM (Fig. S11, ESI†), where the particles were found to be uniform spheres with size ranging 50–500 nm. The bismuth rhombic dodecahedra (Bi RD) were synthesised by reducing bismuth neodecanoate with oleylamine in the presence of 1-octanethiol as a capping agent to control the shape. SEM imaging (Fig. S9, ESI†) displayed the variety of shapes consistent with rhombic dodecahedra in various orientations, and revealed a tight size distribution of 75 ± 5 nm, compared to the 200 nm particles previously reported. While the particles were smaller than intended, the XRD spectrum (Fig. S8, ESI†) displayed the same key feature, the 104 peak slightly higher in intensity than the 110 peak.

The first nanomaterial evaluated was the Bi nanospheres. To test their performance as a catalyst for DECO_2_R, 1 mL of the nanoparticle ink was added to the reaction vessel and dried giving an approximate catalyst loading of 19 mg. Immediately upon addition of the charged mediator solution, a large amount of gas bubbles formed, indicating very high activity, with complete discharge in less than five minutes. Formate constituted one third of the observed products, with the other two thirds being almost entirely hydrogen. The activity of these nanoparticles was much higher than that of the 100-mesh Bi powder, discharging the mediator solution more than ten times faster for a similar mass loading (19 mg *vs.* 25 mg), as would be expected due to the huge increase in surface area. However, the increased activity is compromised by a decrease in both CO_2_R selectivity (33 ± 1.2% *vs.* 54 ± 4.7%) and overall faradaic efficiency (49 ± 5.1% *vs.* 54 ± 0.4%). This does not compare favourably with the previously reported results, where the Bi NS slightly outperformed the bulk Bi in terms of selectivity for formate at a given potential.

The second nanomaterial evaluated was the Bi rhombic dodecahedra (Bi RD). The catalyst was deployed in the reaction vessel by drying 0.5 mL of catalyst ink in the vessel prior to addition of the mediator, for an approximate loading of 15 mg. Following the addition of charged mediator solution, a small number of bubbles began to form, and, after approximately 15 minutes, the reaction appeared to be complete. Product analysis revealed a CO_2_R selectivity of 87 ± 0.2%, with formate making up an impressive 85 ± 0.3% of the observed products. This is in agreement with the previously reported selectivity when the catalyst was deployed in a conventional H-cell at the same operating potential. This successfully established DECO_2_R as a viable technique, albeit constrained by the need for catalysts with high selectivity at relatively mild potentials. Ultimately, performance was limited by the low faradaic yield of just 45%, which remains the biggest drawback of the batch technique. The nanoparticles did not readily disperse in water due to the hydrophobic 1-octanethiol capping agent, resulting in the formation of film like aggregations during the DECO_2_R reaction. SEM imaging of spent catalyst (Fig. S10, ESI†) indicated minimal decomposition of the catalyst after the single pass of charged mediator.

As a practical macro scale catalyst, a Bi foil was evaluated in the same reaction conditions for the sake of comparison. The foil gave the lowest yield of CO_2_R products out of all the Bi materials tested at just 10% of the observed products, with an overall faradaic yield of 36%. The performance of the foil was defined by its low activity, resulting in a discharge time of around seven days, on the same timescale as the observed self-discharge.

### Mechanistic insights

As mentioned, bismuth is known to be highly selective towards formate production when used for ECO_2_R as a conventional heterogeneous electrocatalyst. Bismuth, along with many other catalysts, tends to be most selective towards ECO_2_R at operating potentials around and below −1.0 V *vs.* RHE.^25^ At more positive potentials, there is insufficient energy to activate the thermodynamically and kinetically demanding initial electron insertion into CO_2_ (thermodynamic potential of −1.9 V *vs.* SHE without stabilisation *via* a catalyst), making water reduction to hydrogen the only viable reaction, even on purportedly unfavourable catalysts.

**Fig. 5:**
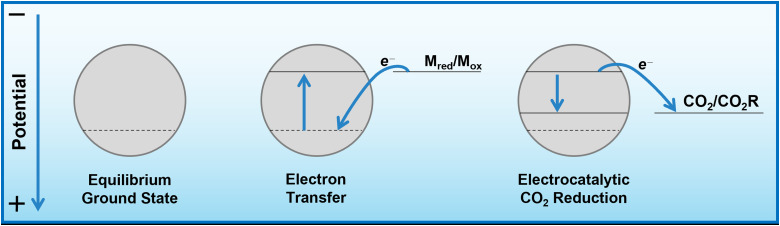
Energy level diagram indicating the change in Fermi level of the catalyst during redox mediated ECO_2_R. When the catalyst is introduced to the charged mediator solution, electrons will be transferred onto the catalyst until its Fermi level is equal to the electrochemical potential of the solution. Upon contact with a reducible species such as CO_2_, electrons of sufficient energy will be used in spontaneous electrochemical reactions, lowering the Fermi level until there are no more electrons with enough energy to react.

An insufficiently negative potential is the primary reason justifying the low product selectivity observed in the DECO_2_R reactions, as ECO_2_R is inhibited by the limited overpotential that the mediator is able to provide, resulting in low activity, leaving hydrogen as the only kinetically feasible product in most cases. As discussed, the Fermi level of the catalyst is controlled and maintained by electron transfer from the redox mediator (Fig. 5). Assuming a fast electron transfer, the Fermi level of the catalyst will be the same as the electrochemical potential of the solution, which itself can be calculated from the Nernst equation for a given state of charge, allowing for estimation of the electrochemical potential at which the reduction reactions are occurring.^31,33^ During the reaction this potential will be controlled by the rate limiting reaction step; either the electron transfer from mediator to catalyst, or more likely the initial electron transfer to CO_2_ by the catalyst.^34^ If the electron transfer is much faster than the reduction of CO_2_, the Fermi level of the catalyst will be that of the solution, while if the CO_2_ reduction step is much faster, the Fermi level of the catalyst will be the thermodynamic potential required for the given product. If the rates are similar, the potential will be somewhere between these two values. As the batch reaction proceeds, the electrochemical potential of the solution will shift as the ratio of reduced to oxidised mediator changes.

It is proposed that Bi favours CO_2_R when deployed in the decoupled reaction not because it is an especially good catalyst for ECO_2_R (high operating potential and low current density compared to many other ECO_2_R catalysts), but because HER is highly disfavoured. The mechanism by which Bi electrochemically reduces CO_2_ is typically assumed to be a predominantly outer-sphere process in which the electron is inserted into a weakly interacting CO_2_ molecule.^21,26,35–37^ This reduced intermediate is not formally adsorbed on the catalyst surface, though it is retained near the surface through weak interactions with the oxygen atom(s). The resulting radical intermediate is highly energetic and will quickly react with a nearby molecule which, in typical aqueous ECO_2_R conditions, is likely to be H_2_O or HCO_3_^−^. An *OCHO intermediate is then formed, which is key to directing the catalyst selectivity towards formate as the major product. The intermediate then undergoes further reduction and HCOO^−^ is released.

The selectivity and low overpotential requirement of the Bi RD is explained as being due to the high degree of shape control in which only two crystal facets are primarily exposed (104 and 110).^22^ These facets are predicted to stabilise the *OCHO intermediate much more than the lowest energy facet (012), and are predicted to have a greater difference between the binding energies of the key CO_2_R and HER intermediates. The high degree of stabilisation of the intermediates drastically reduces the activation energy needed for formate production, lowering the overpotential required for its production, thus improving selectivity by further impeding the competing HER, allowing for high selectivity over a wide potential range.

As a product, formic acid has many industrial uses, and has also been considered as an energy carrier to replace those derived from fossil fuels, both directly in fuels cells and as a hydrogen carrier.^38,39^ Formate/formic acid is typically considered the end of its reduction chain, unlike the other two-electron reduction product, CO, which is considered a key intermediate in the pathways toward the further reduced products observed on copper electrocatalysts.^40^ It has, however, been reported that on select catalysts, formic acid can be reduced to methanol, albeit with low current density and poor selectivity.^41–43^ The primary difficulty with formate/formic acid as a product is the need for intricate separation procedures, which is further complicated in the decoupled system by the redox meditator. It is desirable for the mediator to be indefinitely reusable in a semi-closed system where only CO_2_ and water are added and products are collected. Gaseous products such as H_2_ and CO are optimal, as they spontaneously separate allowing for direct extraction and purification without processing the liquid electrolyte. The same is not true for liquid products, which require invasive extraction techniques to occur as a function within the system to maintain reusability of the mediator and electrolyte. A better understanding of the mechanism in decoupled CO_2_ reduction may enable catalyst design to promote the CO reduction route and deliver gaseous or highly volatile products for ease of separation.

## Conclusions

Decoupled electrochemical CO_2_ reduction to formate has been successfully demonstrated using a range of tailored bismuth catalysts, serving as a valuable proof-of-concept. The single highest yield of a CO_2_ reduction product was 85% (FE 38%) and in all cases the competing HER contributed a significant portion of the products formed. Total faradaic yields ranged between 45–60%, indicating an inefficiency in the experimental design attributed to discharge of the mediator through unquantified pathways that remain to be identified. As discussed, the principal limiting factor for DECO_2_R is the redox potential of the mediator, which is constrained by the water solvent window. Cr PDTA, with its particularly negative redox couple, is able to provide as much driving force as is practically feasible for the reaction, and when paired with a catalyst material that is selective at this low operating potential is able to achieve the same level of selectivity as conventional electrocatalysis.

Further opportunities include widening catalyst scope to improve selectivity and faradaic efficiency, and to enable different products (primarily CO); testing new mediators which may provide improved electron transfer efficiency, a greater driving force (more negative redox potential), and co-catalytic effects such as proton/CO_2_ shuttling. Furthermore, changes in medium, such as moving to a non-aqueous environment, may improve the system by enabling use of redox mediators operating at much more negative potentials, drastically increasing CO_2_ solubility and allowing greater control of proton availability to suppress the competing HER. Ultimately, implementation of DECO_2_R into a flow system will allow the extension of the ‘dual circuit flow battery’ to products other than hydrogen that may be more practical and/or valuable. To this end, the key challenges remain supressing hydrogen evolution and targeting products that are easily extracted.

## Author contributions

M. P., C. G. A. and K. E. T. conceptualised the project. M. P. and C. G. A. synthesised the materials. M. P. performed the experiments and analysed the results, with assistance from D. E. S. in methodology. M. P. wrote the manuscript and all authors contributed to revision.

## Conflicts of interest

There are no conflicts to declare.

## Supplementary Material

EY-002-D3EY00271C-s001
