# Supplementary material for: Electrochemically decoupled reduction of CO2 to formate over a dispersed heterogeneous bismuth catalyst enabled via redox mediators
Source: EES Catal. 2023 Nov 28;2(1):379–88. doi: 10.1039/d3ey00271c (PMC10782805; doi:10.1039/d3ey00271c)
Supplement: EY-002-D3EY00271C-s001 [file EY-002-D3EY00271C-s001.pdf]

Electronic Supplementary Material (ESI) for EES Catalysis.
This journal is © The Royal Society of Chemistry 2023

Supplementary Information

Electrochemically Decoupled Reduction of CO_2 to Formate Over a Dispersed Heterogeneous Bismuth Catalyst Enabled via Redox Mediators

Mark Potter,[a] Daniel E. Smith,[a] Craig G. Armstrong,[a] Kathryn E. Toghill*[a]

[a] Department of Chemistry, Lancaster University, Lancaster, LA1 4YB

* Corresponding author E-mail: k.toghill@lancaster.ac.uk

Contents

Voltammetry 2
UV-Vis Spectroscopy 4
SEM 7
Product Quantification 9
Images 12
References 14

Voltammetry

Unless otherwise stated, cyclic voltammetry was performed in a three-electrode cell with a glassy carbon working electrode (area of 0.0707 cm^2), Ag/AgCl reference electrode (1 M KCl inner solution), and a platinum wire counter electrode at a scan rate of 100 mV s^{-1}.

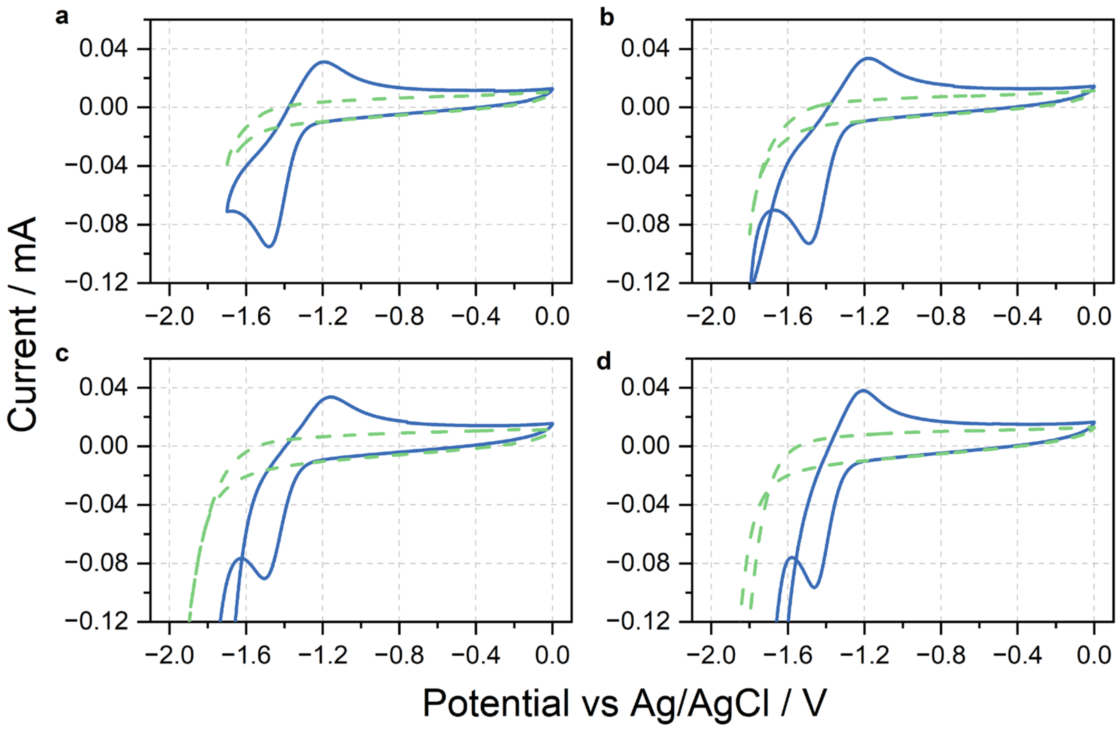

Fig. S1 Cyclic voltammograms of Cr PDTA with $KHCO_3$ supporting electrolyte in water saturated with N_2 at sequentially more negative potential vertices. The electrolyte consisted of 1 M $KHCO_3$ in water with (solid blue) and without (dashed green) 10 mM Cr PDTA target analyte. The negative potential vertex was shifted more negative for each subsequent voltammogram, **a**, −1.7 V, **b**, −1.8 V, **c**, −1.9 V, **d**, −2.0 V.

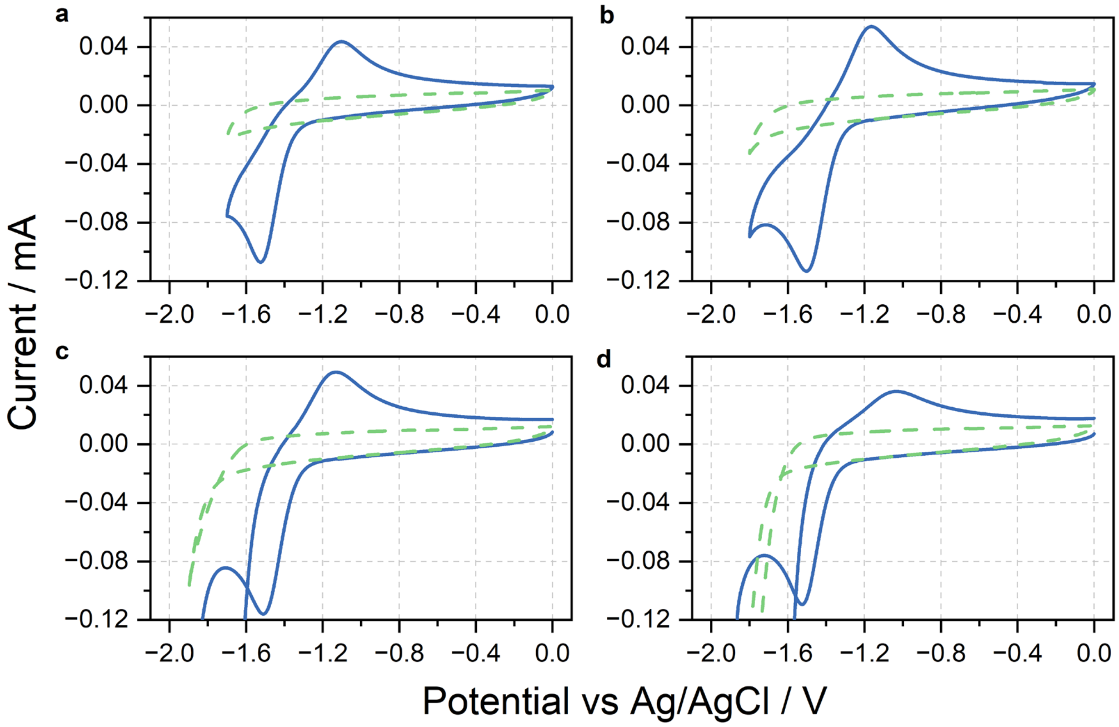

Fig. S2 Cyclic voltammograms of Cr PDTA with KCl supporting electrolyte in water saturated with N_2 at sequentially more negative potential vertices. The electrolyte consisted of 1 M KCl in water with (solid blue) and without (dashed green) 10 mM Cr PDTA target analyte. The negative potential vertex was shifted more negative for each subsequent voltammogram, **a**, −1.7 V, **b**, −1.8 V, **c**, −1.9 V, **d**, −2.0 V.

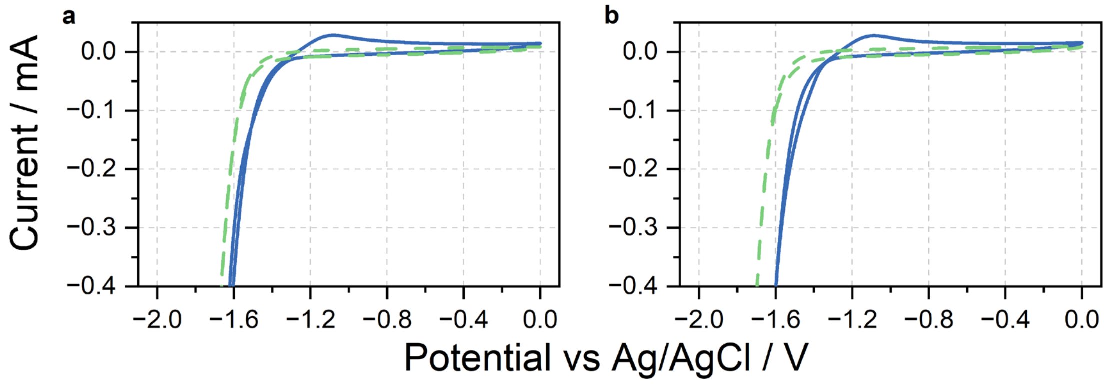

Fig. S3 Cyclic voltammograms of Cr PDTA with $KHCO_3$ supporting electrolyte in water saturated with CO_2 at sequentially more negative potential vertices. The electrolyte consisted of 1 M $KHCO_3$ in water with (solid blue) and without (dashed green) 10 mM Cr PDTA target analyte. The negative potential vertex was shifted more negative for each subsequent voltammogram, **a**, −1.7 V, **b**, −1.8 V.

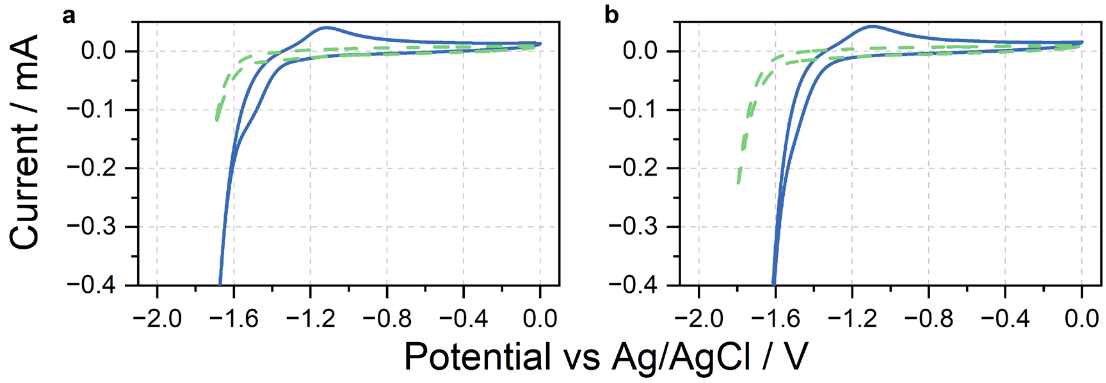

Fig. S4 Cyclic voltammograms of Cr PDTA with KCL supporting electrolyte in water saturated with CO_2 at sequentially more negative potential vertices. The electrolyte consisted of 1 M KCl in water with (solid blue) and without (dashed green) 10 mM Cr PDTA target analyte. The negative potential vertex was shifted more negative for each subsequent voltammogram, **a**, −1.7 V, **b**, −1.8 V.

UV-Vis Spectroscopy

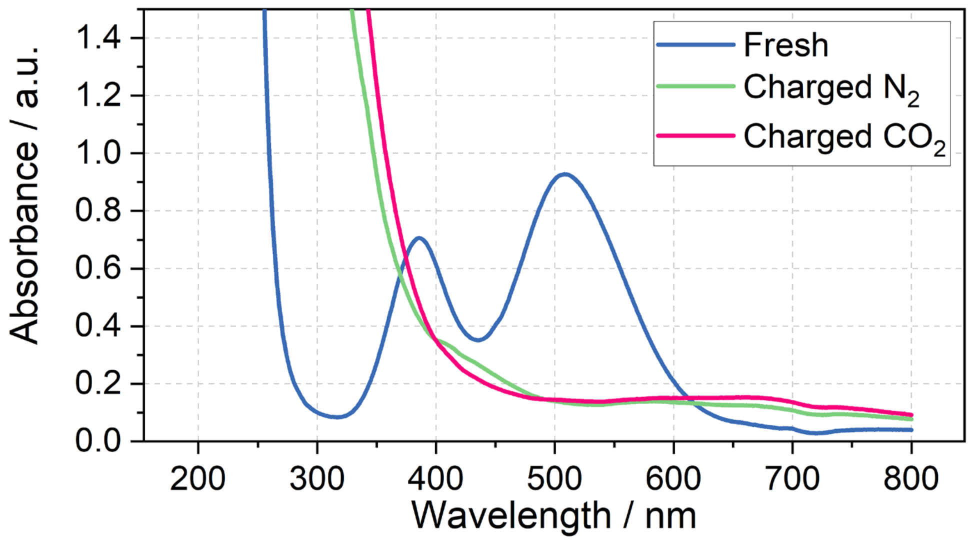

Fig. S5 UV-Vis spectra of Cr PDTA with $KHCO_3$ in its ground and charged states. The samples consisted of 10 mM Cr PDTA with 1 M $KHCO_3$ in water. Spectra were collected from a 10 mm path length quartz cuvette. In its fresh Cr(III) ground state, the complex is a bright red (blue line) with two distinct absorbance peaks at 506 and 383 nm respectively, and an exponential increase in absorbance below 280 nm. When reduced to Cr(II) under an N_2 atmosphere, the complex changes colour to a pale green (green line) which displays no distinct absorbance peaks, with the exponential increase in current now onset around 400 nm. When the atmosphere is replaced with CO_2, the complex visibly changes to a blue-green colour (pink line), however the UV-Vis spectra show minimal change.

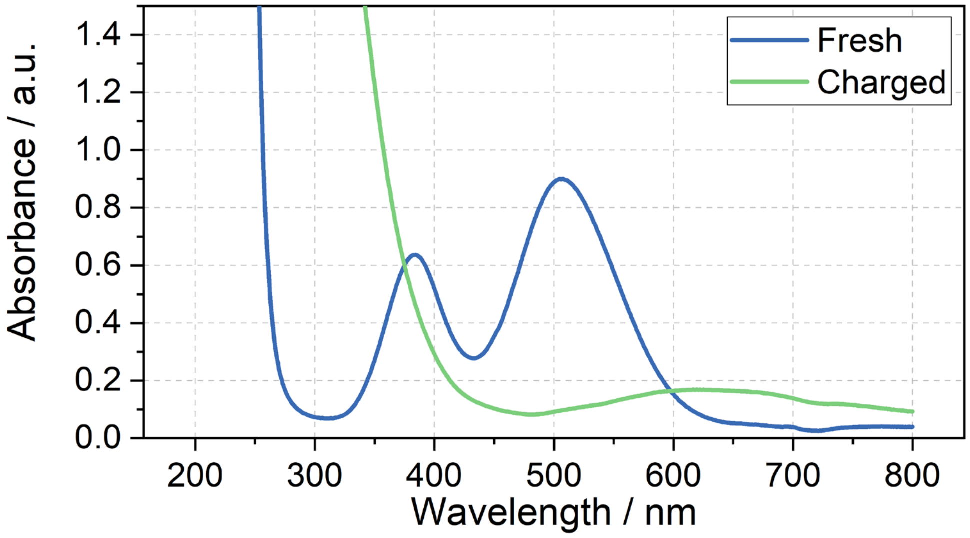

Fig. S6 UV-Vis spectra of Cr PDTA with KCl in its ground and charged state. The samples consisted of 10 mM Cr PDTA with 1 M KCl in water. Spectra were collected from a 10 mm path length quartz cuvette. In its Cr(III) ground state, the complex appears identical to when $KHCO_3$ is present, with the same red colour and absorbance peaks. When reduced to Cr (III), the complex changes to a pale sky-blue colour. Again, the complex displays no distinct absorbance peaks with the spectrum again appearing very similar to when $KHCO_3$ is present. The complex appears identical when either an N_2 or CO_2 atmosphere is used.

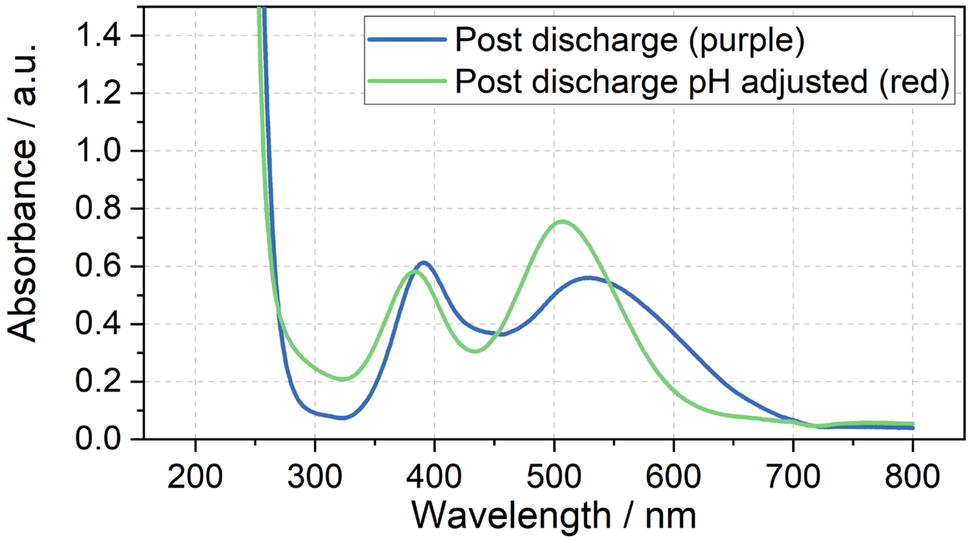

Fig. S7 UV-Vis spectra of used Cr PDTA with KCl. The samples consisted of 10 mM Cr PDTA with 1 M KCl in water. Spectra were collected from a 10 mm path length quartz cuvette. When KCl is used as the supporting electrolyte, mediator solution that has been used in batch decoupled electrolysis does not return to the red ground state as initially expected, instead taking on a purple hue. As purple is also the colour resulting from a mixture of the red Cr(III) and blue Cr(II) states when KCl is present, colour can no longer be used to determine when the reaction is finished. The UV-Vis spectrum of the used purple sample has two peaks in broadly the same positions as the fresh solution, however the higher wavelength peak is broader with lower max absorbance. The solution was found to have a pH of 11, which is much higher than its initial pH of 7.8. Dropwise addition of acid to return the pH to 7.8 restored the red colouration and UV-Vis spectrum of the fresh solution.

XRD

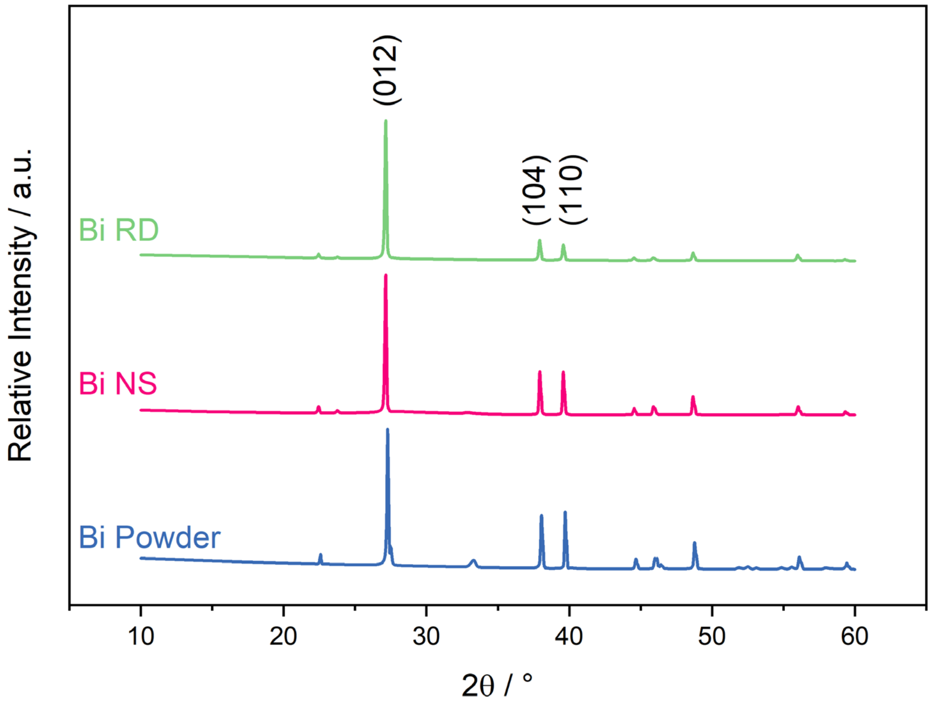

Fig. S8 Powder XRD spectra of the bismuth catalyst materials. Largest peak (012) set to the same intensity for each sample. **Blue**, 100-mesh bismuth powder, **Pink**, solvothermally reduced bismuth nanospheres, **Green**, chemically reduced bismuth rhombic dodecahedra. All three samples contain the expected peaks for the (012), (104) and (110) facets. As the lowest energy facet, (012) is the dominant facet in each sample. The Bi RD are theorised to selectively expose only 104 and 110 facets, while the powder and NS are not expected to selectively expose any particular facets. The key difference between the spectra is the ratio of the (104) and (110) peaks, where the Bi RD favour the (104) facet while the Bi NS does not favour either. This lines up reasonably with the previously reported characterisation of these material.[1]

SEM

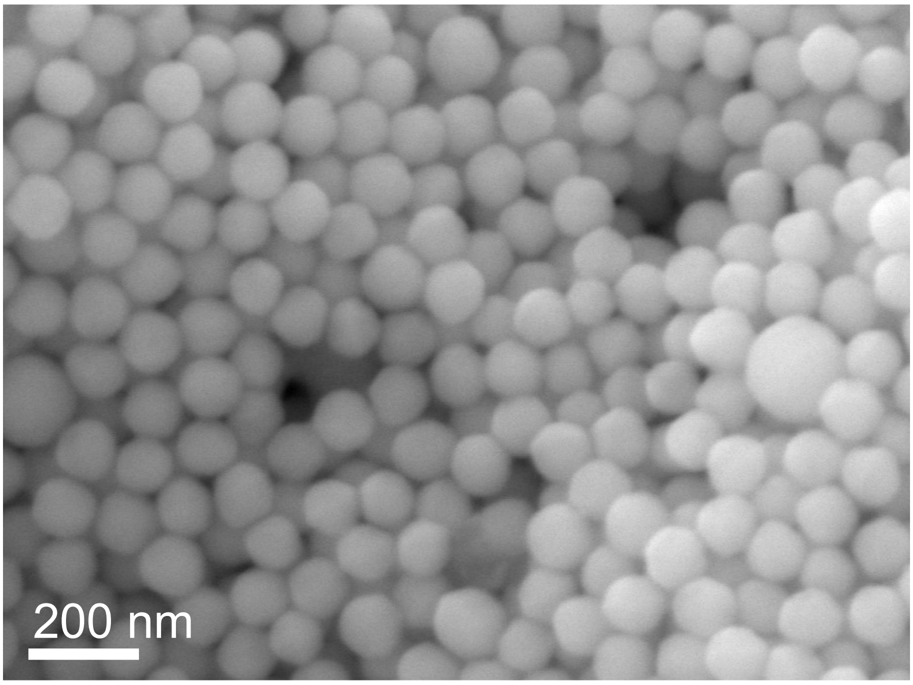

Fig. S9 SEM image of fresh Bi rhombic dodecahedra as synthesised. Scale bar 200 nm. An average particle size of 70-80 nm is observed. The range of shapes, including hexagons and octagons, is representative of rhombic dodecahedra observed from a variety of angle.

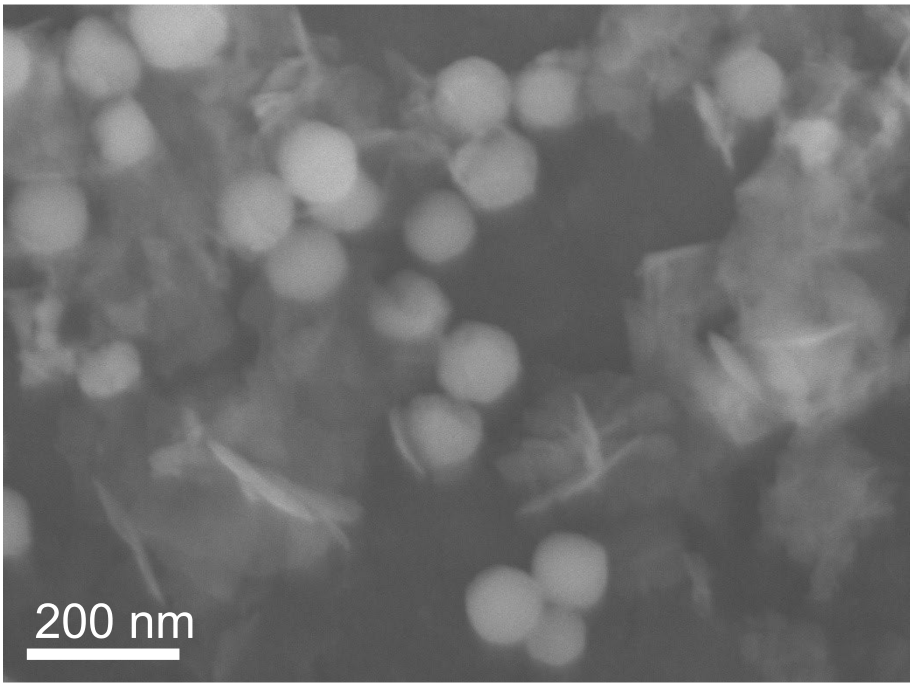

Fig. S10 SEM image of spent Bi rhombic dodecahedra used in mediated CO_2 reduction. Sample collected post discharge with 200 nm syringe filter and washed with water and ethanol, some salt remained. Nanoparticles have retained their size and shape post catalysis.

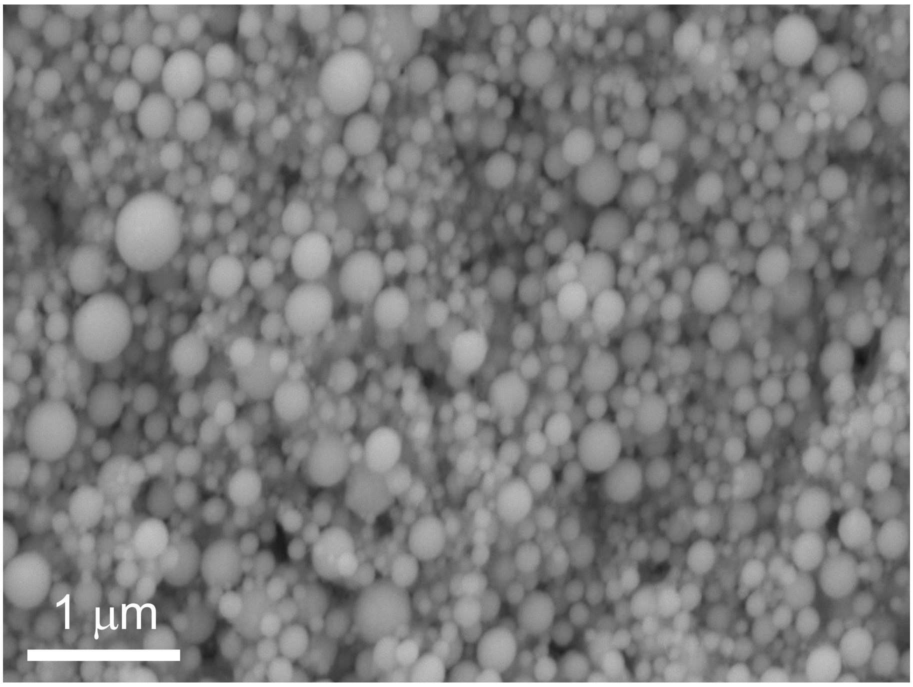

Fig. S11 SEM image of fresh Bi nanospheres as synthesised. Scale bar 1 μm. Particles are observed as uniform spheres with sizes ranging 50-500 nm.

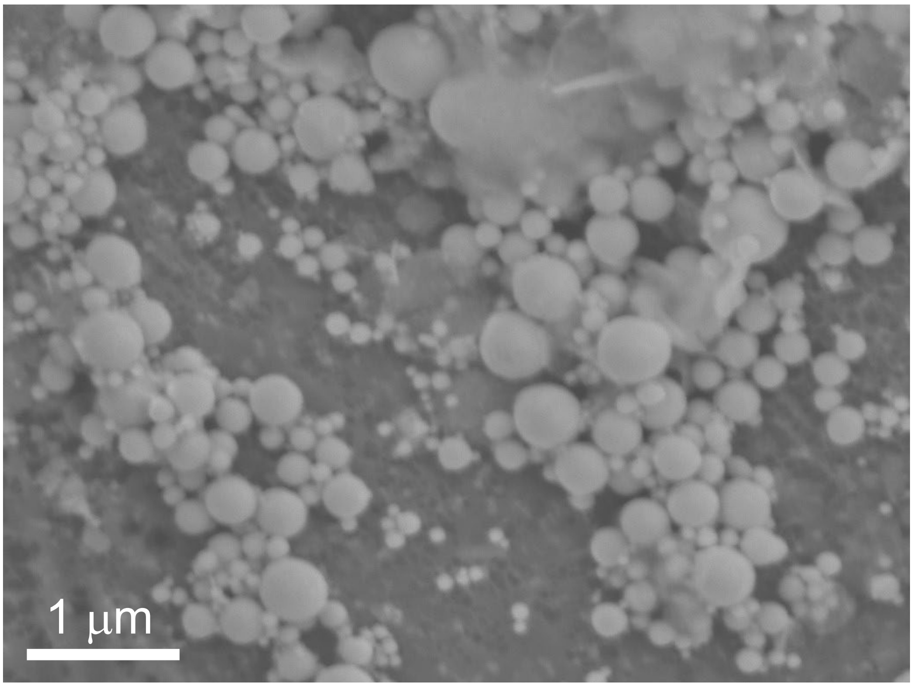

Fig. S12 SEM image of spent Bi nanospheres used in mediated CO_2 reduction. Scale bar 1 μm. Sample collected post discharge with 200 nm syringe filter and washed with water, some salt remained. Particle shape and size unaltered by use as catalyst.

Product Quantification

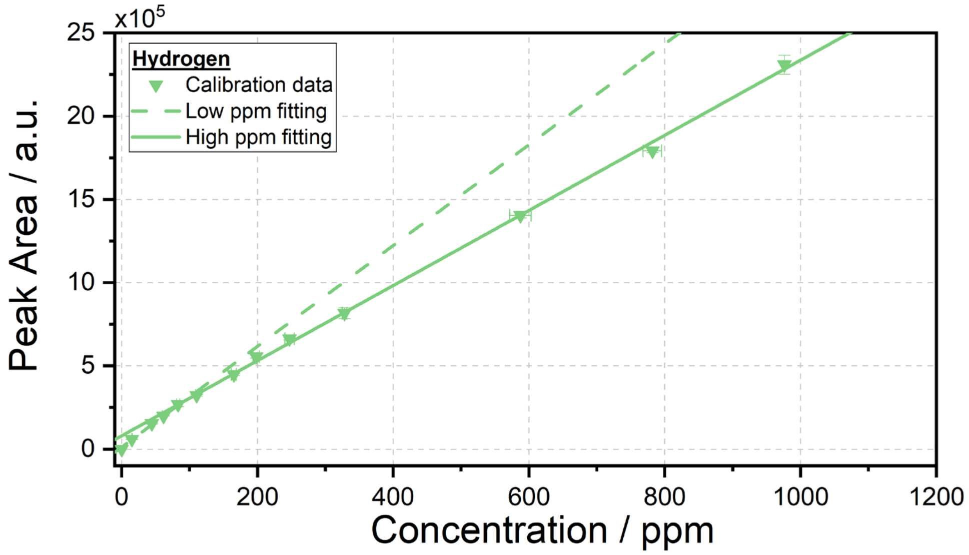

Fig. S13 Gas chromatography calibration for hydrogen concentration with high and low concentration fittings. Samples were prepared from a calibration standard containing 976 ppm H_2, 1009 ppm CH_4, 1040 CO, and 1018 ppm C_2H_4, with He balance. Dilutions were performed in a gas tight pressure vessel with He to give concentrations of approximately 1000, 800, 600, 336, 250, 166, 112, 84, 62, 44, ang 15 ppm.

Equations in the form y = mx + c used to determine the concentration of H_2 in a real sample from the GC peak area;

$$\text{Peak area} = (3028.7 \times \text{ppm}) + 12335$$

For the low concentration range 10-100 ppm, and;

$$\text{Peak area} = (2257.2 \times \text{ppm}) + 80578$$

For the high concentration range 100-1000 ppm.

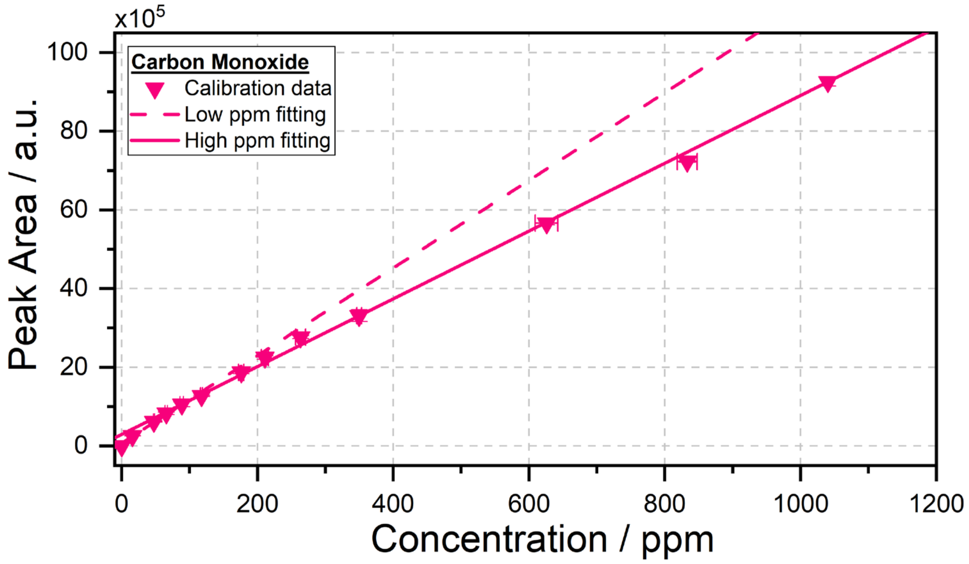

Fig. S14 Gas chromatography calibration for carbon monoxide concentration with high and low concentration fittings. Samples were prepared from a calibration standard containing 976 ppm H_2, 1009 ppm CH_4, 1040 CO, and 1018 ppm C_2H_4, with He balance. Dilutions were performed in a gas tight pressure vessel with He to give concentrations of approximately

1000, 800, 600, 336, 250, 166, 112, 84, 62, 44, ang 15 ppm. The low calibration range 10-100 ppm and the high calibration range 100-1000 ppm have strong correlation, allowing for high confidence in the accuracy of determined concentrations.

Equations in the form y = mx + c used to determine the concentration of CO in a real sample from the GC peak area;

$$\text{Peak area} = (11141 \times \text{ppm}) + 70204$$

for the low concentration range 10-100 ppm, and;

$$\text{Peak area} = (8615.8 \times \text{ppm}) + 29657$$

for the high concentration range 100-1000 ppm.

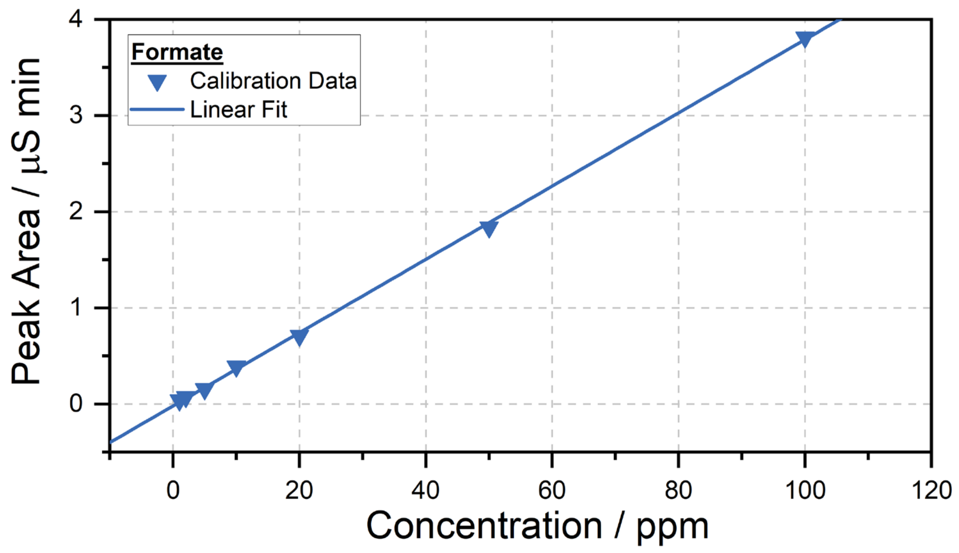

Fig. S15 Ion chromatography calibration for $HCOO^-$ concentration in ppm. Samples were prepared from a calibration standard containing 1000 ppm $HCOO^-$ by dilution with ultrapure water to concentrations of 100, 50, 20, 10, 5, 2, and 1 ppm. The calibration is strongly correlated, allowing for a high degree of certainty in the accuracy of determined concentrations.

An equation in the form y = mx + c was used to determine the concentration of $HCOO^-$ in a real sample from the IC peak area;

$$\text{Peak area} = (0.038070 \times \text{ppm}) + 0.018176$$

Gaseous Products. To determine the faradaic efficiency for the gaseous products H_2 and CO, headspace gas was taken from the flask and analysed by gas chromatography. An aliquot of gas was collected in a gas tight syringe and diluted with additional background gas to ensure the products were within the calibration range (5 or 10 mL of sample diluted into 50 mL for five- and ten-fold dilutions respectively). The sample was then directly injected into the GC inlet for separation. Using the above calibration plots, the concentration of each product within the diluted sample was determined in ppm (molar ratio). This can then be multiplied by the dilution factor to estimate the concentration of each product within the vessel headspace. It is assumed that 100% of all gaseous products were in the headspace, as both H_2 and CO are sparing soluble in water and the electrolyte was degassed via sonication immediately before sampling.

The amount of each gaseous product in moles within the headspace was estimated;

$$\textit{Moles product [mol]} = \frac{\textit{Headspace concentration [ppm]}}{10^6} \times \textit{Moles of gas in headspace [mol]}$$

where the ideal gas law was used to determine the total number of moles within the headspace;

$$\textit{Total moles of gas in headspace} = \frac{\textit{Headspace pressure [Pa]} \times \textit{Headspace volume [m}^3\textit{]}}{\textit{Ideal gas constant [J K}^{-1}\textit{ mol}^{-1}\textit{]} \times \textit{Temperature [K]}}$$

By considering the electron stoichiometry, the amount of charge needed to form each product can be calculated;

$$\textit{Charge required [C]} = \textit{Moles product [mol]} \times \textit{Electron stoichiometry} \times \textit{Faraday constant [C mol}^{-1}\textit{]}$$

which can then be compared to the total charge passed;

Charge passed [C] =

Concentration of mediator [M] × Volume of mediator [L] × Electron stoichiometry × Faraday constant [C mol^{-1}]

to determine the faradaic efficiency as a quantum yield;

$$\text{Faradaic efficiency} = \frac{\text{Charge required [C]}}{\text{Charge passed [C]}} \times 100$$

Liquid Products. To determine the faradaic efficiency for the liquid product $HCOO^-$, a portion of the electrolyte was analysed by ion chromatography. By use of the above calibration, the ppm (w/w) of $HCOO^-$ within the sample could be estimated. From this, the total moles produced can be calculated (assuming a sample density of 1 Kg L^{-1});

$$\text{Moles product [mol]} = \frac{\text{Measured Concentration [ppm]}}{\text{Molecular mass } [\text{Kg mol}^{-1}]} \times \text{Total volume of electrolyte [L]}$$

which can then be used to determine the charge required;

$$\text{Charge required [C]} = \text{Moles product [mol]} \times \text{Electron stoichiometry} \times \text{Faraday constant [C mol}^{-1}]$$

which can then be compared to the total charge passed;

Charge passed [C] =

Concentration of mediator [M] × Volume of mediator [L] × Electron stoichiometry × Faraday constant [C mol^{-1}]

to determine the faradaic efficiency as a quantum yield;

$$\text{Faradaic efficiency} = \frac{\text{Charge required [C]}}{\text{Charge passed [C]}} \times 100$$

Images

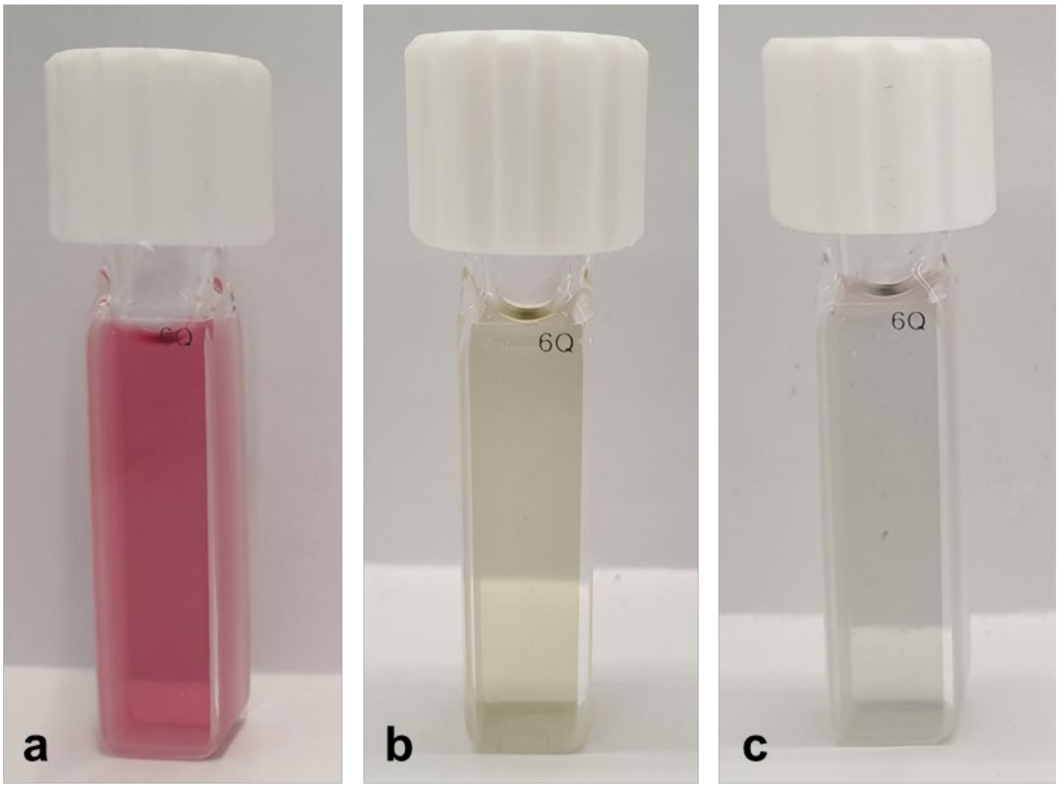

Fig. S16 10 mM Cr PDTA with 1 M $KHCO_3$ in water. **a**, red ground state. **b**, green reduced state under N_2. **c**, blue-green reduced state under CO_2.

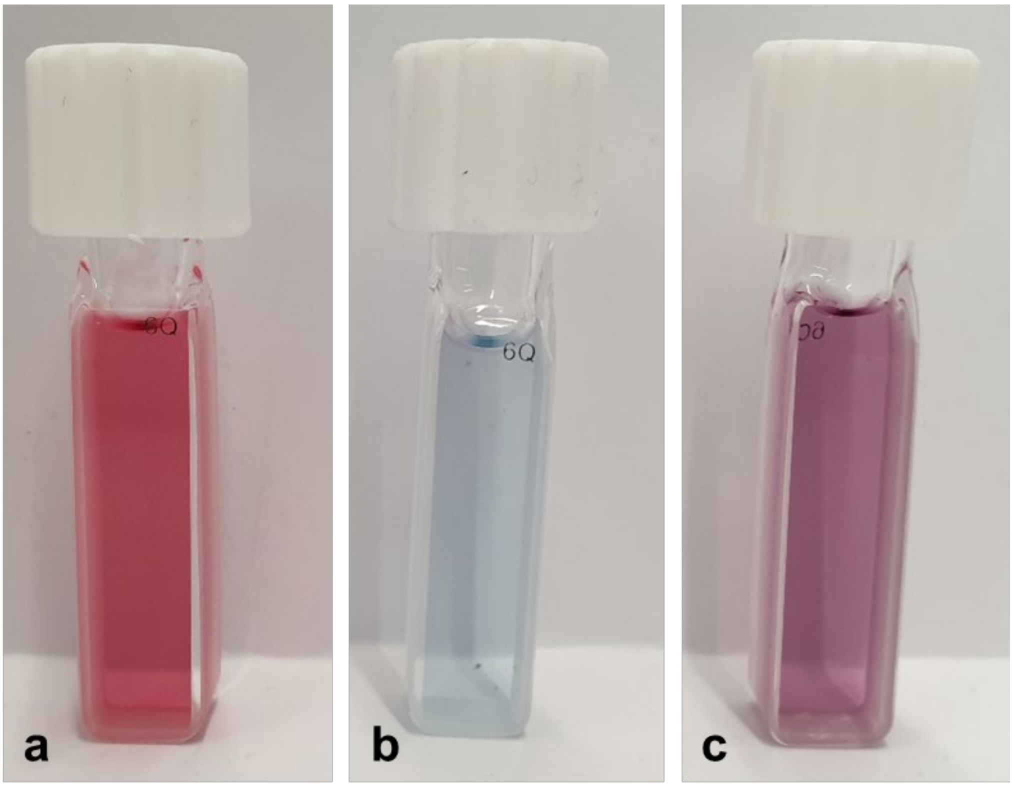

Fig. S17 10 mM Cr PDTA with 1 M KCl in water. **a**, red ground state. **b**, blue reduced state. **c**, high pH purple discharged state.

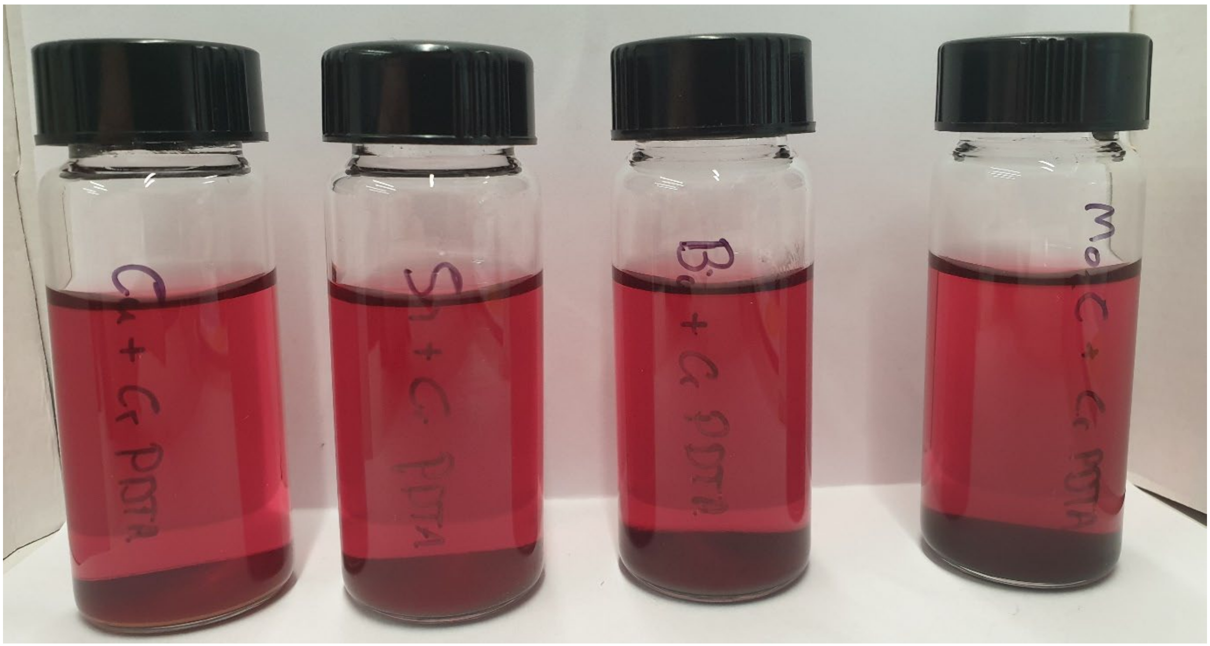

Fig. S18 Cr PDTA stability testing. Samples of 10 mM Cr PDTA with 1 M $KHCO_3$ in water with (left to right) Cu, Sn, Bi and Mo_2C powder immediately after preparation.

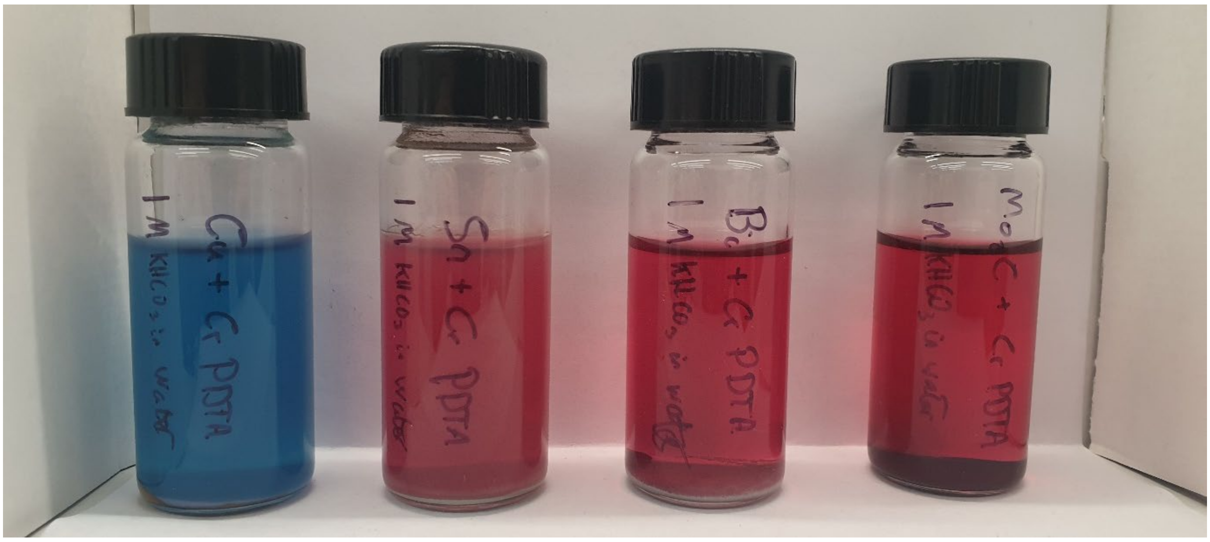

Fig. S19 Cr PDTA stability testing. Samples of 10 mM Cr PDTA with 1 M $KHCO_3$ in water with (left to right) Cu, Sn, Bi and Mo_2C powder after three months. The copper powder has displaced the chromium from the complex, resulting in a deep blue Cu(II) solution and plating the copper particles light grey with chromium. The Sn powder has plated the glass vial however the complex is unaffected. Both bismuth and molybdenum carbide underwent no visible reaction with the complex.

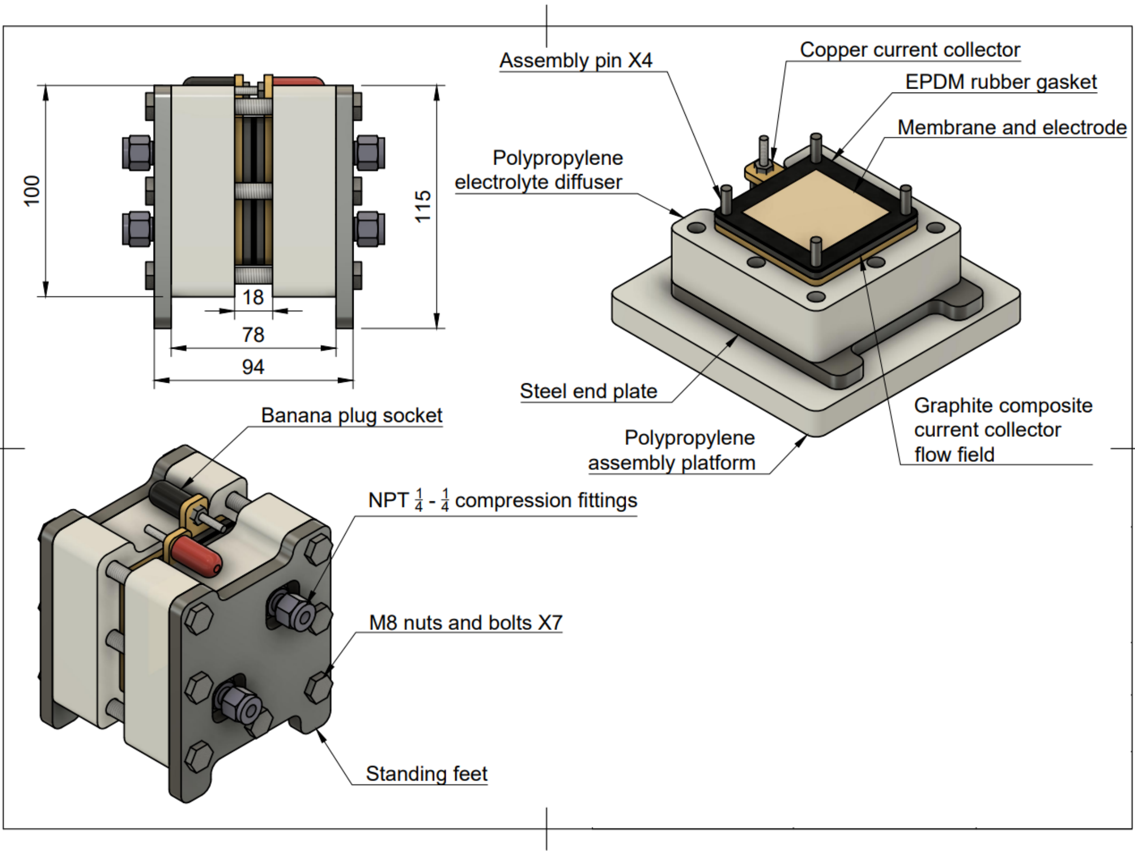

Fig. 20 Custom Flow Cell. Schematic of the custom 16 cm^2 electrode area flow cell used to charge the redox mediator.

References

1 H. Xie, T. Zhang, R. Xie, Z. Hou, X. Ji, Y. Pang, S. Chen, M. M. Titirici, H. Weng and G. Chai, *Adv. Mater.*, 2021, **33**, 1–10.
